# Towards Enhanced MRI Performance of Tumor-Specific Dimeric Phenylboronic Contrast Agents

**DOI:** 10.3390/molecules26061730

**Published:** 2021-03-19

**Authors:** Jonathan Martinelli, Lorenzo Tei, Simonetta Geninatti Crich, Diego Alberti, Kristina Djanashvili

**Affiliations:** 1 Department of Biotechnology, Delft University of Technology, Van der Maasweg 9, 2629 HZ Delft, The Netherlands; jonathan.martinelli@uniupo.it; 2 Department of Science and Technological Innovation, Università del Piemonte Orientale, Viale Michel 11, 15121 Alessandria, Italy; lorenzo.tei@uniupo.it; 3 Department of Molecular Biotechnology and Health Sciences, University of Torino, Via Nizza 52, 10126 Torino, Italy; simonetta.geninatti@unito.it (S.G.C.); diego.alberti@unito.it (D.A.)

**Keywords:** gadolinium complexes, MRI contrast enhancement, phenylboronic acid, sialic acid, tumor targeting

## Abstract

It is known that phenylboronic acid (PBA) can target tumor tissues by binding to sialic acid, a substrate overexpressed by cancer cells. This capability has previously been explored in the design of targeting diagnostic probes such as Gd- and ^68^Ga-DOTA-EN-PBA, two contrast agents for magnetic resonance imaging (MRI) and positron emission tomography (PET), respectively, whose potential has already been demonstrated through in vivo experiments. In addition to its high resolution, the intrinsic low sensitivity of MRI stimulates the search for more effective contrast agents, which, in the case of small-molecular probes, basically narrows down to either increased tumbling time of the entire molecule or elevated local concentration of the paramagnetic ions, both strategies resulting in enhanced relaxivity, and consequently, a higher MRI contrast. The latter strategy can be achieved by the design of multimeric Gd^III^ complexes. Based on the monomeric PBA-containing probes described recently, herein, we report the synthesis and characterization of the dimeric analogues (Gd^III^-DOTA-EN)_2_-PBA and (Gd^III^-DOTA-EN)_2_F_2_PBA. The presence of two Gd ions in one molecule clearly contributes to the improved biological performance, as demonstrated by the relaxometric study and cell-binding investigations.

## 1. Introduction

**Figure 1:**
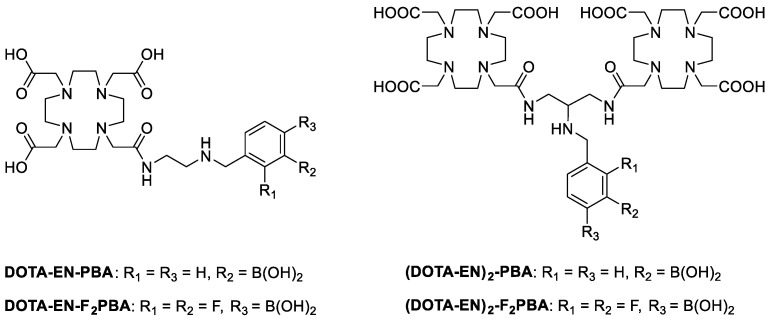
Structures of monomeric (DOTA-EN)- and dimeric (DOTA-EN)_2_-phenylboronic compounds.

Boronic acids continue to find wider applications in biomedical research, material science, chemical synthesis and electronics [1]. In particular, the recent interest in phenylboronic acid (PBA) for diagnostic and therapeutical purposes has arisen from its ability to bind sialic acid (SA), a nine-carbon monosaccharide unit that is overexpressed as the terminal group of glycolipids and glycoproteins on the surface of tumor cells [2], thus making them exploitable in the design of targeting contrast agents. PBA derivatives can reversibly form five- or six-membered cyclic boronate esters with the exocyclic polyol chain of SA [3], and this property has already been exploited in the preparation of a few diagnostic targeting probes [4,5,6]. Among them, Gd^III^-DOTA-EN-PBA has proven particularly successful as a magnetic resonance imaging (MRI) contrast agent for in vivo tumor targeting based on the recognition of overexpressed sialic acid [7]. This Gd^III^ complex bears a PBA group conjugated to a polyamino-polycarboxylic ligand for paramagnetic ions (DOTA = 1,4,7,10-tetraazacyclododecane-1,4,7,10-tetraacetic acid) through an aminoethylamide spacer (EN = ethylenediamine) (Figure 1). It was proven that a two-site cooperative binding mechanism takes place: on one side, the previously mentioned formation of a cyclic boronate between the B(OH)_2_ moiety and the diol of the substrate; on the other side, recognition is enhanced by an electrostatic interaction between the negatively charged carboxylate on the SA residue and the positively charged amine of the linker between the macrocycle and PBA. The success of Gd^III^-DOTA-EN-PBA targeting led to further investigation of this ligand with the radioactive ^68^Ga^III^ ion, enabling visualization of tumors by positron emission tomography (PET) [8]. Very recently, a paper was published by our group reporting a double improvement regarding this type of functionalized ligand: a more efficient synthetic procedure and an enhancement in binding capability [9]. Concerning the former issue, it is well-known that the amphiphilic character of boronates causes difficulties in their preparation and purification, including solubility problems, tendency to form oligomers or react with nucleophilic species, and the pH dependence of the equilibrium between a boronic acid and its boronate [10]. In our synthetic approach, we exploited solid-phase synthesis to overcome all these problems by binding the boronic site to a suitable functionalized support (DEAM-PS resin) [11,12,13] for temporary protection of the moiety, thus obtaining a final cleaner product at a higher yield [9]. This procedure was then successfully applied to prepare an analogous ligand modified at the phenyl ring with the introduction of two electron-withdrawing fluorine atoms in order to increase the acidity of the phenylboronic group and, consequently, its ability to bind SA residues on the surface of tumor cells. The switch of the boronate from the *meta*-position in DOTA-EN-PBA to the *para*-position in the novel DOTA-EN-F_2_PBA allowed testing of the importance of the formation of the cyclic boronate in the recognition mechanism, regardless of the additional contribution of the previously mentioned amine-carboxylate electrostatic interaction. The cell binding experiments that were carried out with the Gd^III^-DOTA-EN-F_2_PBA complex confirmed the improved affinity that counterbalanced the loss of the electrostatic effect.

Herein, we report a further advancement in the DOTA-EN-PBA-based ligands consisting of their dimeric analogues (DOTA-EN)_2_-PBA and (DOTA-EN)_2_-F_2_PBA (Figure 1). Due to the intrinsic low sensitivity of MRI, the preparation of dimers or multimers (e.g., tetramers, hexamers, etc.) has become a common strategy to improve the performance of MRI contrast agents by increasing the local concentration of paramagnetic ions and the global tumbling time (*τ*_R_) of the agent. A high relaxivity density (i.e., high relaxivity per Gd ion) usually implies a significant enhancement of the relaxation rate per mass unit of MRI agent, often referred to as mass relaxivity [14], which represents an important parameter in molecular imaging applications. On the other hand, an increased molecular size leads to a lengthening in *τ*_R_ and, in turn, a higher longitudinal relaxivity *r*_1_. Examples of ditopic DOTA-like systems were reported by using Gd^III^-DOTA monoamide [15,16], Gd^III^-HPDO3A [17], and Gd^III^-DOTA [18]. The relaxivity enhancement of these systems as compared to monomeric Gd complexes was often very much dependent on the water exchange rate or on the rigidity of the linker. The two novel ligands reported in the present work, still prepared by exploiting the proven advantages of solid-phase synthesis, are endowed with two DOTA-macrocycles connected via EN-linkers to a phenylboronic acid or a difluorophenylboronic acid, respectively. The two resulting DOTA monoamide (DOTAMA) macrocycles in each ligand are well known to form thermodynamically and kinetically stable chelates with Gd^III^ ions [19].

For consistency in the comparison, the positions of the boronic acid moiety and the fluorine atoms with respect to the aromatic ring were maintained exactly the same as in the monomeric ligands. The corresponding Gd^III^ complexes of both chelates were then prepared and fully characterized through standard ^1^H and ^17^O NMR relaxometric techniques. Finally, the targeting abilities and contrast enhancement of the novel dimeric (Gd^III^-DOTA-EN)_2_-F_2_PBA complex were investigated on suitable tumor cell cultures, i.e., cell cultures overexpressing a sufficient amount of SA.

## 2. Results and Discussion

### 2.1. Synthesis

**Scheme 1:**
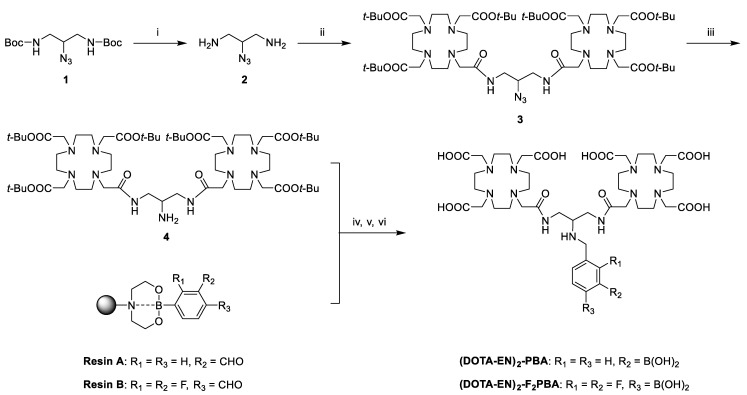
Synthesis of (DOTA-EN)_2_-PBA and (DOTA-EN)_2_-F_2_PBA: (**i**) 4 M HCl, dioxane, rt, 15 h; (**ii**) DOTA(Ot-Bu)_3_, HBTU, HOBt, DIPEA, DMF, rt, 15 h; (**iii**) H_2_, Pd/C, rt, 3 h; (**iv**) THF, rt, 3 h; (**v**) NaBH_4_, THF, rt, 4 h; (**vi**) TFA, DCM, rt, 1 h.

The first part of the synthesis of the (DOTA-EN)_2_-ligands (Scheme 1) started with the preparation of *N*,*N*′-di-Boc-2-azido-1,3-diaminopropane **1** (Boc = *tert*-butoxycarbonyl protecting group) according to a reported procedure [20]. The amino groups of 1,3-diamino-2-propanol were protected with di-*tert*-butyl dicarbonate and then the hydroxyl group was activated as the corresponding mesylate and converted into an azide.

The two primary amines of **1** were deprotected by stirring the compound in 4 M HCl, leading to compound **2** as hydrochloride salt that was reacted directly with tris-*tert*-butyl-protected DOTA in the presence of peptide coupling agents to obtain the dimer **3**. The DOTA-precursor was, in turn, prepared converting commercial DO3A hydrobromide salt into its monoethyl ester [7] that was hydrolyzed under strong alkaline conditions into the corresponding carboxylic acid. The azide moiety of **3** was then reduced to amine, leading to the product **4**, which was used in a reductive amination with the suitable formylphenylboronic acid previously immobilized on DEAM-PS resin. Final cleavage and deprotection with TFA afforded the desired compounds. The characterization of the intermediates and the final products was performed via IR and NMR spectroscopies (Figures S1–S3). (DOTA-EN)_2_-PBA and (DOTA-EN)_2_-F_2_PBA were obtained with a final 32 and 36% yield, respectively, and the part of the process involving a boronate moiety was easily carried out thanks to the previously mentioned advantages of solid-phase synthesis. Such results confirm the validity of the synthetic protocol recently reported by our group for the preparation of this type of functionalized ligand [9].

**Scheme 2:**
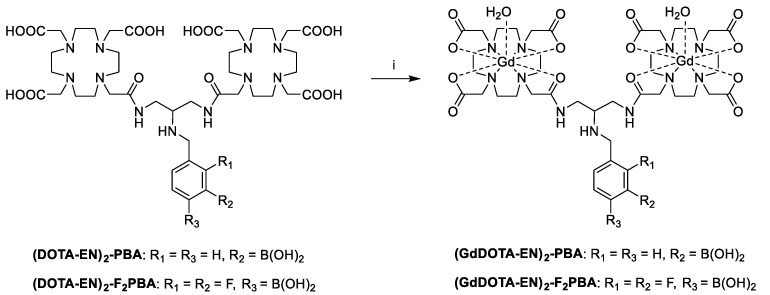
Synthesis of (Gd^III^-DOTA-EN)_2_-PBA and (Gd^III^-DOTA-EN)_2_-F_2_PBA: i) GdCl_3_·6H_2_O, pH 7, 50 °C, 15 h.

The corresponding Gd^III^ complexes (Gd^III^-DOTA-EN)_2_-PBA and (Gd^III^-DOTA-EN)_2_-F_2_PBA were prepared by mixing each ligand with a slight excess of GdCl_3_ hexahydrate (Scheme 2). The unreacted Gd^III^ was precipitated as hydroxide at pH 10 and filtered off. The formation of the complexes was confirmed by ESI^+^ mass spectrometry (Figures S4 and S5). The paramagnetic complexes obtained upon lyophilization were used directly to investigate their properties in terms of performance as MRI contrast agents and targeting probes. The stability of the novel probes can be considered similar to the monomeric analogue Gd-DOTA-EN-PBA such as it has been found in transmetallation studies with endogenous Zn^II^ ions in the presence of phosphate buffer (Figure S6) [21].

### 2.2. Relaxometric Characterizations

The longitudinal relaxivity value *r*_1_ (per 1mM Gd) at 25 °C and 20 MHz of the binuclear complexes is 8.7 mM^−1^ s^−1^ for (Gd^III^-DOTA-EN)_2_-PBA and 8.2 mM^−1^ s^−1^ for (Gd^III^-DOTA-EN)_2_-F_2_PBA, with a ca. 40% increase compared to the values measured for the mononuclear species (6.2 and 5.8 mM^−1^ s^−1^, respectively) [9]. The relaxivity enhancement can be easily attributed to a longer value of the rotational correlation time associated with the larger molecular size, combined with the retention of the same hydration state (*q* = 1) for each metal ion. As has already been noticed for the monomeric complexes [9], the fluorinated chelate has a slightly lower relaxivity: this can probably be ascribed to a decrease in its hydrophilicity induced by the fluorine atoms on the aromatic ring that affects the hydration of the molecule and then the rotational dynamics. The obtained relaxivity values are in agreement with a *q* = 1 hydration state, as confirmed by all DOTA monoamide-related studies, since the first paper about Gd^III^-DOTAMA was reported almost 30 years ago [15].

**Figure 2:**
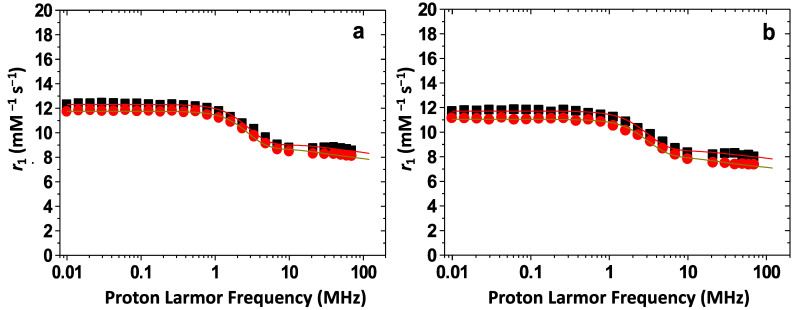
Comparison between the ^1^H NMRD profiles acquired at pH 7 and 25 (■) or 37 °C (●) for aqueous solutions of (Gd^III^-DOTA-EN)_2_-PBA (**a**) and (Gd^III^-DOTA-EN)_2_-F_2_PBA (**b**).

**Figure 3:**
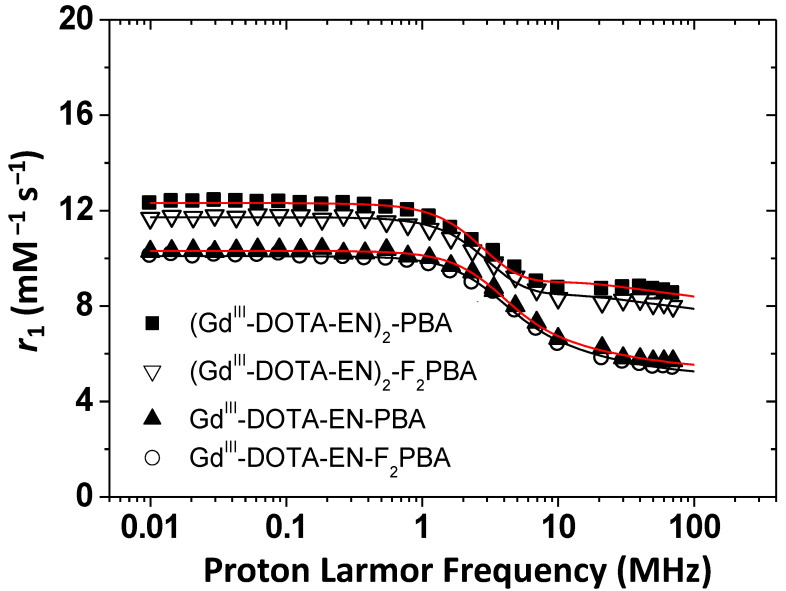
Comparison between the ^1^H NMRD profiles of aqueous solutions of (Gd^III^-DOTA-EN)_2_-PBA, (Gd^III^-DOTA-EN)_2_-F_2_PBA, Gd^III^-DOTA-EN-PBA and Gd^III^-DOTA-EN-F_2_PBA. All profiles were acquired at 25 °C and pH 7. The solid lines represent the best-fitting results of the experimental data points.

The relaxometric characterization of each binuclear Gd^III^ complex was carried out by recording their ^1^H nuclear magnetic relaxation dispersion (NMRD) profiles in the frequency range 0.01 to 70 MHz (2.34 × 10^−4^ − 1.64 T) at 25 and 37 °C (Figure 2). The *r*_1_ values at 37 °C are only slightly lower than those measured at 25 °C, over the entire range of investigated proton Larmor frequencies, indicating that the water exchange rate (*k*_ex_ = 1/*τ*_M_) might be slow. In Figure 3, the NMRD profiles at 25 °C of the two binuclear compounds are compared with the two mononuclear analogues. For the Gd^III^ dimers a broad peak is observable at higher magnetic fields (centered at 40 MHz), typical of multimeric species and due to the effects induced by the increased molecular weight of the whole molecule on the rotational correlation time (*τ*_R_) of each complex (see below).

It is noteworthy that the relaxivities of (Gd^III^-DOTA-EN)_2_-PBA and (Gd^III^-DOTA-EN)_2_-F_2_PBA calculated per concentration of dimer are 17.4 and 16.3 mM^−1^ s^−1^, respectively. These values imply that, in order to achieve the same MRI contrast enhancement as obtained with the corresponding monomers, it is sufficient to use a molar amount of such agents about 36% of that required for Gd^III^-DOTA-EN-PBA or Gd^III^-DOTA-EN-F_2_PBA.

**Figure 4:**
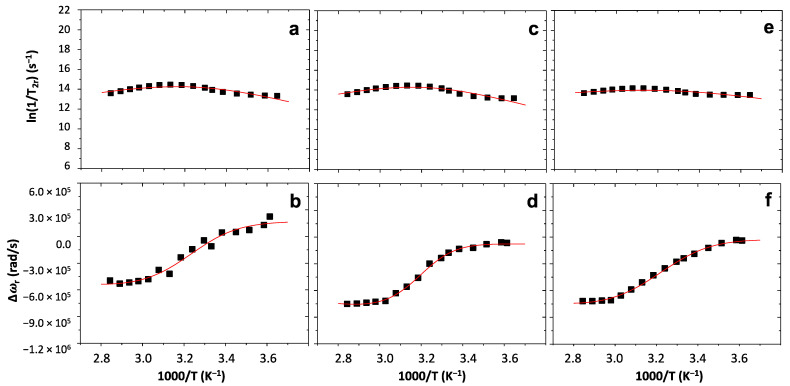
Reduced transverse ^17^O relaxation rates (**top**) and chemical shifts (**bottom**) measured at 11.7 T and pH 7 for Gd^III^-DOTA-EN-PBA (**a**,**b**), Gd^III^-DOTA-EN-F_2_PBA (**c**,**d**) and (Gd^III^-DOTA-EN)_2_-F_2_PBA (**e**,**f**). The solid lines represent the best-fitting results of the experimental data points.

First, a complete ^1^H and ^17^O NMR relaxometric study was carried out on the monomers to obtain a more accurate analysis of the water exchange dynamics of the Gd^III^ complexes. Thus, the accurate value of the water exchange time *τ*_M_ (Table 1) was obtained by analyzing the temperature dependence of the ^17^O NMR transverse relaxation rate (1/T_2_) and the paramagnetic shift (Δω) of the solvent water at 11.7 T. The experimental data, reported as reduced transverse relaxation rates (1/T_2r_) and reduced chemical shifts (Δω_r_), are shown in Figure 4. The ^1^H NMRD and ^17^O NMR data were fitted simultaneously, according to the established theory of paramagnetic relaxation. Thus, while NMRD data were analyzed using the standard Solomon–Bloembergen–Morgan model for the inner-sphere relaxation mechanism [22,23,24] and Freed’s model for the outer-sphere components [25], the temperature dependence of 1/T_2r_ and Δω was evaluated by using the Swift–Connick equations [26,27] (see details in the Supplementary Materials). Due to the number of parameters implicated in the fitting procedure, some of them were fixed to known or plausible values, namely the hydration number (*q*), the Gd^III^-H_2_O distance (*r*), the distance of closest approach of the outer sphere water molecules to Gd^III^ (*a*) and the relative diffusion coefficient at 25 °C (*D*). The temperature dependence of *τ*_V_ (correlation time for the modulation of the transient zero-fit splitting, ZFS) and *τ*_R_ has been considered through their activation energies *E*_V_ (set to 1 kJ mol^−1^) and *E*_R_ (fixed to 10 kJ mol^−1^). The fit was carried out using *τ*_R_, Δ^2^ (trace of the ZFS), *τ*_V_, *τ*_M_, the enthalpy of activation Δ*H*_M_ and the scalar Gd-^17^O_w_ coupling constant *A/ħ* as adjustable parameters. The best-fit parameters for Gd^III^-DOTA-EN-PBA and Gd^III^-DOTA-EN-F_2_PBA (Table 1) show values in line with those reported for other Gd^III^-DOTA monoamides [19,28], both in terms of electronic parameters (Δ^2^and *τ*_V_) and of water exchange dynamics. In particular, *τ*_M_ values of 471 ns for Gd^III^-DOTA-EN-PBA and 486 ns for Gd^III^-DOTA-EN-F_2_PBA were determined, slightly lower than those reported for other Gd^III^-DOTA monoamides [19,28,29]. Finally, the shape of the 1/T_2r_ plot is further confirmation of a *q* = 1 hydration state.

Although no variation in the water exchange parameters was expected passing from the mononuclear to the binuclear complexes, ^17^O NMR data were acquired also for the fluorinated dimer to confirm this hypothesis (Figure 4). Therefore, the simultaneous fitting of the ^17^O NMR and ^1^H NMRD data for (Gd^III^-DOTA-EN)_2_-F_2_PBA and the fitting of the NMRD profiles at 25 and 37 °C for (Gd ^III^-DOTA-EN)_2_-PBA were carried out using the same equations and procedure described above, resulting in the parameters shown in Table 1. For the binuclear complexes, the higher molecular weight (MW = ca 1000 Da) implies a slower rotational dynamic of the paramagnetic probes compared to the monomeric analogues (MW = ca 600 Da), mirrored by the values of *τ*_R_ (ca 100 ps for the monomers vs. ca 200 ps for the dimers). As mentioned above, this increase in *τ*_R_ is responsible for the broad peak observed in the NMRD profiles at higher magnetic field strengths. Unfortunately, the intermediate/long *τ*_M_ values (around 0.5 μs), typical of Gd^III^-DOTA monoamide complexes, limit the *r*_1_ increase from mononuclear to binuclear complexes to only 40%, lower than that observed when the water residence lifetime is lower than 100 ns (fast exchange regime) [30].

### 2.3. Determination of the Binding Efficacy

**Figure 5:**
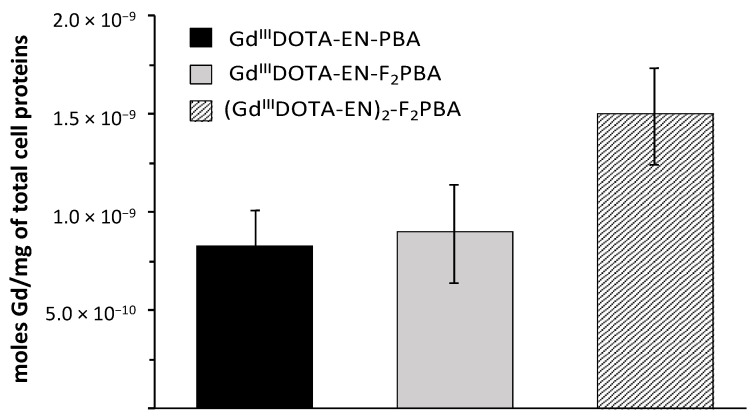
Evaluation of cell binding of Gd complexes upon their incubation with melanoma B16-F10 cells for 4 h in EBSS buffer w/o glucose at a concentration of 0.6 mM Gd. Error bars indicate ± SD and the mean values are obtained from three independent experiments.

Considering the combined improvements (i.e., the two paramagnetic ions and the higher acidity of the boronic moiety), (Gd^III^-DOTA-EN)_2_-F_2_PBA represents the most promising dimeric MRI agent, and therefore, it was chosen for the evaluation tests on the ability of cell binding. The initial investigation was conducted on murine melanoma B16-F10 cells, in order to compare the results directly with the previous experiments on the monomeric species [9]. While the cell binding of Gd^III^-DOTA-EN-PBA and Gd^III^-DOTA-EN-F_2_PBA was comparable, as was expected, the amount of gadolinium internalized by cells incubated in the presence of Gd dimer is significantly higher due to the 2:1 ratio between Gd^III^-chelates and PBA in each molecule (Figure 5). It is possible that the presence of two chelates increases the polarity of that side of the probe, thus partially affecting its internalization by cells, and explaining why the measured Gd amount is less than twice the amount of the monomers (ca 1.5-fold).

**Figure 6:**
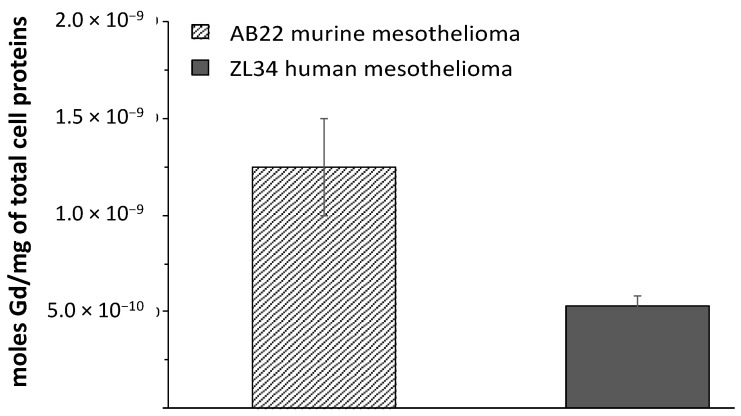
Evaluation of the bound (Gd^III^-DOTA-EN)_2_-F_2_PBA complex upon incubation with AB22 (murine) and ZL34 (human) mesothelioma cells: 4 h incubation in EBSS buffer w/o glucose at a concentration of 0.6 mM Gd. Error bars indicate ±SD and the mean values are obtained from three independent experiments.

A study that has recently been reported in the literature highlights that mesothelioma cells express a high amount of SA on their surface [31,32,33]. Mesothelioma is the malignant neoplasm of the mesothelial cells and is a highly aggressive tumor lacking any significant therapies. Thus, it was decided to implement a binding test of (Gd^III^-DOTA-EN)_2_-F_2_PBA with two mesothelioma cell lines (ZL34 human and AB22 murine). The results presented in Figure 6 show a high amount of cell bound fluorinated Gd dimer that, in the case of AB22, was similar to that found for the B16 melanoma. Since there is no evidence in the literature for a distinctive SA expression by mesothelioma cells of various species, the difference in binding found for AB22 and ZL34 can be explained by the intrinsic variability of SA in these tumors.

**Figure 7:**
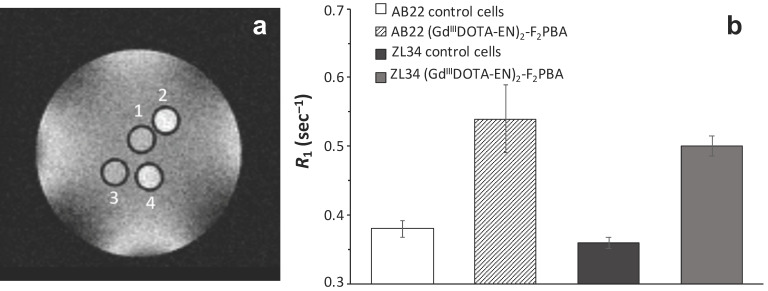
T_1W_ image (TR = 250 ms, TE = 3.8 ms) of a phantom containing glass capillaries filled with AB22 murine mesothelioma cells (control, 1) incubated with 0.6 mM (Gd^III^-DOTA-EN)_2_-F_2_PBA (2) and ZL34 human mesothelioma cells (control, 3) incubated with 0.6 mM (Gd^III^-DOTA-EN)_2_-F_2_PBA (4) (**a**) and their corresponding values of longitudinal relaxivities (**b**). Error bars indicate ±SD and the mean values are obtained from three different slices.

Finally, a T_1_-weighted MR image of AB22 and ZL34 mesothelioma cells was acquired (Figure 7a) and their corresponding *R*_1_ was measured (Figure 7b) showing that the amount of internalized Gd is sufficient to generate a positive contrast, thus demonstrating that the probe can be used to detect mesothelioma cells.

## 3. Materials and Methods

### 3.1. General Remarks

All chemicals were purchased from Sigma-Aldrich Co. (Merck, St. Louis, MO, USA), Alfa Aesar Co. (Thermo Fisher GmbH, Kandel, Germany) and Acros Organics and were used without further purification. “H_2_O” refers to high purity water with conductivity of 0.04 μS cm^−1^, obtained from a “Milli-Q” purification system. Bio-Rad AG 501-X8 resin was preliminarily washed with water and methanol. Thin-layer chromatography (TLC) was carried out on silica plates (silica gel 60 F254, Merck 5554, Darmstadt, Germany) and visualized by UV lamp (254 nm) or stained in KMnO_4_, ninhydrin, bromocresol green or curcumin solution as appropriate. Preparative column chromatography was carried out using silica gel (Merck Silica Gel 60, 230 ± 400 mesh) pre-soaked in the starting eluent.

^1^H, ^13^C, ^11^B and ^19^F NMR spectra (including ^1^H-decoupled ^13^C NMR) and homo-/hetero-nuclear bidimensional maps were recorded on a Bruker Avance III spectrometer (Bruker, Milano, Italy) operating at 11.74 T, corresponding to a protonic resonance frequency of 499.8 MHz. Samples were prepared in 5 mm NMR tubes by dissolving the compounds in appropriate deuterated solvents. Chemical shifts are reported in ppm relative to TMS (0 ppm for ^1^H and ^13^C NMR), H_3_BO_3_ (0 ppm for ^11^B NMR) and CF_3_COOH (−74.4 ppm for ^19^F NMR) as internal standards. For ^11^B NMR experiments, a quartz tube was used. Splitting patterns are described as singlet (s), broad singlet (bs), doublet (d), double doublet (dd), triplet (t), multiplet (m) or broad multiplet (bm). Coupling constants are reported in Hz.

ESI mass spectra were recorded on a Waters SQD 3100. Analytical HPLC-MS was carried out on a Waters modular system equipped with Waters 1525 binary pump, Waters 2487 UV/Vis and Waters SQD 3100 (ESCI ionization mode) detectors, using an XBridge^TM^ Phenyl 3.5 μm 4.6 × 150 mm column (Waters Corporation, Milford, MA, USA).

The water proton longitudinal relaxation rates as a function of the magnetic field strength were measured in non-deuterated aqueous solutions on a Fast Field-Cycling Stelar SmarTracer relaxometer (Stelar s.r.l., Mede (PV), Italy) over a continuum of magnetic field strengths from 0.00024 to 0.25 T (corresponding to 0.01–10 MHz proton Larmor frequencies). The relaxometer operates under computer control with an absolute uncertainty in 1/T_1_ of ± 1%. Additional longitudinal relaxation data in the range 20–70 MHz were obtained on a Stelar Relaxometer connected to a Bruker WP80 NMR electromagnet adapted to variable-field measurements. The exact concentration of Gd^III^ was determined by measurement of bulk magnetic susceptibility shifts of a *t*BuOH signal or by inductively coupled plasma mass spectrometry (ICP-MS, Element-2, Thermo-Finnigan, Rodano (MI), Italy). Sample digestion was performed with concentrated HNO_3_ (70%, 2 mL) under microwave heating at 160 °C for 20 min (Milestone MicroSYNTH Microwave lab station equipped with an optical fiber temperature control and HPR-1000/6M six-position high-pressure reactor, Bergamo, Italy). The ^1^H T_1_ relaxation times were acquired by the standard inversion recovery method with typical 90° pulse width of 3.5 μs and 16 experiments with 4 scans. The temperature was controlled with a Stelar VTC-91 airflow heater equipped with a calibrated copper constantan thermocouple (uncertainty of ±0.1 °C).

Variable-temperature ^17^O NMR measurements were recorded on a Bruker Avance III spectrometer (11.7 T) equipped with a 5 mm probe and standard temperature control unit. Aqueous solutions of the complexes (10–20 mM) containing 2% of the ^17^O isotope (Cambridge Isotope) were used. The observed transverse relaxation rates were calculated from the signal width at half-height.

MR images of capillaries filled with a suitable sample were acquired at 21 °C, 7 T on a Bruker Avance 300 spectrometer equipped with a microimaging probe. T_1W_ images were acquired by using a standard MSME (Multi-Slice Multi-Echo) sequence with the following parameters: TR = 50 ms, TE = 3.3 ms, FOV = 1 × 1 cm, slice thickness = 1 mm, matrix size 128 × 128.

### 3.2. Synthesis

#### 3.2.1. Synthesis of Precursors

##### 1,3-Diamino-2-azidopropane dihydrochloride (**2**)

Boc-diamine **1** [20] (2.74 g, 8.70 mmol) was dissolved in 4 M HCl (30 mL) and dioxane (30 mL) and stirred at room temperature overnight. The solvents were then evaporated and the obtained amine hydrochloride salt (1.36 g, 83%) was used without further purification. 

^1^H NMR (400 MHz, 25 °C, CD_3_OD): 4.27 (m, 1H, CH), 3.33 (dd, *J*^2^ = 13.5 Hz, *J*^3^ = 3.9 Hz, 2H, CHH’), 3.08 (dd, *J*^2^ = 13.5 Hz, *J*^3^ = 8.9 Hz, 2H, CHH’). ^13^C NMR (100 MHz, 25 °C, CD_3_OD): 58.9 (NH-CH_2_-CHN_3_-CH_2_-NH), 41.80 (NH-CH_2_-CHN_3_-CH_2_-NH).

##### DOTA(O*t*-Bu)_3_

The DO3A monoethyl ester [7] (1.16 g, 1.93 mmol) was dissolved in 1:1 H_2_O/MeOH (20 mL) and LiOH (510 mg, 21.29 mmol) was added. The solution was stirred at room temperature overnight. Brine (30 mL) was added, and the water layer was extracted with DCM (2 × 20 mL). The organic phase was dried over anhydrous Na_2_SO_4_, filtered and evaporated to obtain the DOTA derivative 16 as a white solid (410 mg, 37%).

^1^H NMR (400 MHz, 25 °C, CDCl_3_): 2.0–3.5 (bm, ring and acetates CH_2_), 1.31 (s, 27H, CH_3_).

##### (DOTA(Ot-Bu)_3_)_2_-1,3-diamido-2-azidopropane (**3**)

Diaminoazide **2** hydrochloric salt (100 mg, 0.53 mmol) was suspended in DMF (5 mL), DIPEA (0.92 mL, 5.28 mmol) was added, and the suspension was stirred at room temperature for 30 min. Separately, a solution of DOTA(Ot-Bu)_3_ (607 mg, 1.06 mmol), HOBt (143 mg, 1.06 mmol) and HBTU (402 mg, 1.06 mmol) in DMF (5 mL) was prepared and added to the previous suspension. The mixture was stirred at room temperature overnight. The solvent was then evaporated under reduced pressure, the residue was suspended in DCM (50 mL) and washed with brine (3 × 25 mL). The organic layer was dried over anhydrous NaSO4, filtered and evaporated to obtain the product as a yellowish solid (532 mg, 82%).

^1^H NMR (400 MHz, 25 °C, CDCl_3_): 6.55 (m, 2H, NH), 4.11 (m, 1H, CH), 3.45–1.88 (bm, 52H, DOTA ring and acetates + CH_2_CH), 1.41 (s, 54H, CH_3_). ^13^C NMR (100 MHz, 25 °C, CDCl_3_): 172.8 (COO), 165.8 (CONH), 81.8 (CCH_3_), 61.1 (CH), 55.4 (CH_2_CO), 52 + 47 (br, CH_2_ ring), 51.8 (CH_2_CH), 27.7 (CH_3_). IR: 3432, 2974, 2830, 2116, 1727, 1653, 1505.

##### (DOTA(Ot-Bu)_3_)_2_-1,3-diamido-2-aminopropane (**4**)

Compound **3** (450 mg, 0.37 mmol) was dissolved in MeOH (10 mL); 10% Pd/C (45 mg) was added and H_2_ was bubbled for 10 min. The suspension was then stirred overnight under a H_2_ atmosphere. After filtration through Celite, the solvent was removed under vacuum leading to the product as a white solid (378 mg, 85%).

^1^H NMR (400 MHz, 25 °C, CDCl_3_): 6.59 (m, 2H, NH), 4.16 (m, ^1^H, CH), 3.49–1.95 (bm, 52H, DOTA ring and acetates + CH_2_CH), 1.72 (bm, 2H, NH_2_), 1.45 (s, 54H, CH_3_). ^13^C NMR (100 MHz, 25 °C, CDCl_3_): 172.8 (COO), 165.9 (CONH), 81.8 (CCH_3_), 55.7 (CH_2_CO), 54.6 (CH), 52 + 48 (br, CH_2_ ring), 51.9 (CH_2_CH), 27.9 (CH_3_). IR: 3500, 2976, 2829, 1722, 1653, 1538, 872.

#### 3.2.2. General Procedure to Bind Formylphenylboronic Acids to DEAM-PS Resin

DEAM-PS resin (306 mg, 0.51 mmol) and a suitable PBA (0.68 mmol) were weighed in a filter-syringe. Dry THF (5 mL) was added, and the reactor was shaken for 2 h. The suspension was then filtered and washed with dry THF (3 × 5 mL).

#### 3.2.3. Reductive Amination between 4 and PBA-DEAM-PS Resin

PBA-DEAM-PS resin (54 mg, 0.09 mmol) was weighed in a filter-syringe and suspended in dry THF (5 mL). Amine **4** (103 mg, 0.09 mmol) was dissolved in dry THF (2 mL) and added to the reactor. The suspension was shaken for 3h and it was filtered and washed with dry THF (2 × 5 mL). The resulting functionalized resin was resuspended in dry THF (5 mL), NaBH_4_ (7 mg, 0.18 mmol) was added, and the mixture was shaken for 4h. The resin was then filtered and washed with dry DMF (3 × 3 mL), dry DCM (5 × 3 mL) and dry THF (3 × 3 mL).

#### 3.2.4. Cleavage and Deprotection of (DOTA-EN)_2_-PBA and (DOTA-EN)_2_-F_2_PBA

DCM (2 mL) and TFA (2 mL) were added to the resin in the syringe and the suspension was shaken overnight. After filtration and washing with TFA (3 × 3 mL), the combined filtrates were evaporated under reduced pressure and the products were obtained as white solids.

##### (DOTA-EN)_2_-PBA

Yield 32%, 29 mg (0.029 mmol). ^1^H NMR (400 MHz, 25 °C, D_2_O): 7.83 (bs, 1H, CH^Ar^), 7.52 (bm, 2H, CH^Ar^), 6.95 (bm, 1H, CH^Ar^), 3.7–2.8 (bm, 53H, DOTA ring and acetates + CH_2_CHCH_2_). ^11^B NMR (128 MHz, 25 °C, D_2_O): 0.019.

##### (DOTA-EN)_2_-F_2_PBA

Yield 36%, 33 mg (0.032 mmol). ^1^H NMR (400 MHz, 25 °C, CDCl_3_): 7.46 (bm, 1H, CH^Ar^), 7.31 (bm, 1H, CH^Ar^), 3.8–3.0 (bm, 53H, DOTA ring and acetates + CH_2_CHCH_2_). ^11^B NMR (128 MHz, 25 °C, D_2_O): 0.019. ^19^F NMR (376 MHz, 25 °C, D_2_O): −132.86 (d, *J*^3^ = 21.2 Hz, CFCB), −146.97 (d, *J*^3^ = 21.2 Hz, CFCCH_2_).

#### 3.2.5. General Procedure for the Preparation of Gd^III^ complexes

The PBA-ligand (0.032 mmol) was dissolved in H_2_O (10 mL). GdCl_3_·6H_2_O (17 mg, 0.045 mmol) was added and the mixture was stirred overnight at pH 7 and room temperature. The pH was increased to ×≈ 10 by dropwise addition of 0.1 M NaOH, and the resulting precipitate was filtered off. The pH of the filtrate was then returned to 7 by dropwise addition of 0.1 M HCl.

#### 3.2.6. Cell Lines and Incubation Protocol

Mouse melanoma (B16-F10) cell lines were purchased from the American Type Culture Corporation. Melanogenic B16-F10 cells were obtained by growing cells in standard DMEM (Lonza) medium supplemented with 3.7 mg/mL sodium bicarbonate, 4 mM glutamine and 10% FBS (*v*/*v*). Mouse mesothelioma (AB22) cell lines were obtained from Sigma-Aldrich and they were cultured in RPMI medium (Lonza, Basel, Switzerland) supplemented with 25 mM Hepes, 10% (*v*/*v*) FBS and 2mM glutamine. Human mesothelioma (ZL34) cell line was obtained from Sigma Aldrich. The cells were cultured in DMEM–Ham’s F12 (Lonza) containing 2.5 mM glutamine and supplemented with 15% (*v*/*v*) FBS. All media contained 100 U/mL penicillin and 100 U/mL streptomycin. All the cell lines were maintained in a humidified incubator at 37 °C, 5% CO_2_.

For the cell binding experiments, 1.4 × 10^6^ B16-F10, 4.5 × 10^5^ ZL34 and 3 × 10^5^ AB22 were seeded in T25 flasks. After 48 h for B16-F10 and ZL34 and after 24 h for AB22, the cells were incubated with 0.6 mM Gd (Gd^III^-DOTA-EN)_2_-F_2_PBA in EBSS (Earle’s Balanced Salts Solution) *w*/*o* glucose for 4 h at 37 °C, 5% CO_2_. All the incubations were performed at 37 °C, 5% CO_2_. At the end of the incubation, cells were washed three times with 3 mL ice-cold PBS and detached with 1mM EDTA. ZL34 and AB22 cells were also transferred into glass capillaries for MRI analysis. Cells were collocated in glass capillaries inside an agar phantom and MR images were acquired using a standard T_1_-weighted multi slice spin echo sequence (TR (repetition time)/TE (echo time)/NEX number of excitations) = 250/3.7/6, FOV (field of view) = 1.2 cm) on a Bruker Avance300 spectrometer (7T) provided with a Micro 2.5 microimaging probe (Bruker BioSpin, Ettlingen, Germany). T_1_ relaxation times were determined using a standard Saturation Recovery Spin Echo. Finally, all cell samples were transferred in falcon tubes and sonicated at 30% of power for 30 s in ice. The Gd content of each cell sample lysate was determined by the ICP-MS technique (see below). The milligrams of proteins, proportional to the number of cells, of each cell sample lysate, were evaluated by the Bradford assay (BioRad, Hercules, CA, USA) using bovine serum albumin as a standard.

#### 3.2.7. Inductively Coupled Plasma Mass Spectrometry (ICP-MS)

Gd content from cell samples was determined using inductively coupled plasma mass spectrometry (ICP-MS) (Element-2; Thermo-Finnigan, Rodano (MI), Italy). Sample digestion was performed using a high-performance Microwave Digestion System (ETHOS UP Milestone, Bergamo, Italy) after the addition of concentrated HNO_3_ (70%) to cell lysates (1:1), in a final volume of 0.4 mL. The calibration curve was obtained using four Gd absorption standard solutions (Sigma-Aldrich) in the range 0.1–0.004 μg/mL.

## 4. Conclusions

In this work, the synthesis and characterization of two novel dimeric ligands and their Gd^III^ complexes is reported. Both (DOTA-EN)_2_-PBA and (DOTA-EN)_2_-F_2_PBA (improvement of previously reported monomeric analogous species) bear a phenylboronic acid that is meant to be exploited to target sialic acid, overexpressed by cancer cells. The solid-phase approach allowed obtaining of ligands at good yields and with very simple work-up processes. The corresponding Gd complexes were prepared according to a standard procedure and their relaxometric properties were evaluated by recording the respective NMRD profiles and by studying the temperature dependence of the ^17^O NMR transverse relaxation rate and the paramagnetic shift of the solvent water. As expected, the higher Gd content and the bigger size (translating into a longer rotational correlation time *τ*_R_) led to an average 40% improvement in the relaxivity values for (Gd^III^-DOTA-EN)_2_-PBA and (Gd^III^-DOTA-EN)_2_-F_2_PBA (8.71 and 8.16 mM^−1^ s^−1^, respectively at 20 MHz and 25 °C) compared to those previously reported for the corresponding monomeric analogues (6.24 and 5.80 mM^−1^ s^−1^, respectively). This translates, in principle, into a much lower amount (about 36%) of dimeric Gd-chelates that is necessary to administer in order to obtain the same MRI contrast enhancement compared to the corresponding Gd monomers. By fitting the experimental data for both monomeric and dimeric species, the main relaxometric parameters were determined, including the water exchange time *τ*_M_ (ca. 470 ns) and the rotational correlation time *τ*_R_ that, as anticipated, is about double for the Gd dimers (ca. 200 ps) with respect to the monomers (ca. 100 ps) due to the higher molecular weight.

Finally, the binding abilities of (Gd^III^-DOTA-EN)_2_-F_2_PBA were investigated by comparing its internalization by melanoma and mesothelioma tumor cells: in both cases, a good amount of Gd was detected inside the cells upon incubation, which was also sufficient to produce a significant contrast effect in phantom MR images. Therefore, the reported complexes, and particularly (Gd^III^-DOTA-EN)_2_-F_2_PBA, improved in terms of both the number of chelates (two Gd^III^-DOTA units) and the acidity of the boronic moiety (because of the presence of two electron-withdrawing halogen atoms), were confirmed to represent a promising way to develop efficient targeting contrast agents for various diagnostic techniques such as MRI, PET and SPECT. Moreover, the presence of one boron atom on (Gd^III^-DOTA-EN)_2_-F_2_PBA opens perspective for its use as a boron carrier for Boron Neutron Capture Therapy (BNCT). This alternative radiotherapy, thanks to its ability to hit the tumor with a selectivity at the cellular level, is particularly effective in the treatment of diffuse tumors such as mesothelioma [34,35,36].

## Figures and Tables

**Table 1:** Selected parameters obtained from the analysis of the 1/T_1_ NMRD profiles at 298 K and the ^17^O NMR data ^a^.

Parameter	Gd^III^-DOTA-EN-PBA ^b^	Gd^III^-DOTA-EN-F_2_PBA ^b^	(Gd^III^-DOTA-EN)_2_-PBA	(Gd^III^-DOTA-EN)_2_-F_2_PBA
^20MHz^*r*_1p_ [mM^−1^ s^−1^]	6.2 ± 0.1	5.8 ± 0.1	8.7 ± 0.1	8.2 ± 0.1
*τ*_R_ [ps]	109 ± 9	102 ± 2	212 ± 2	203 ± 2
Δ² [10^19^ s^−2^]	1.9 ± 0.1	2.1 ± 0.1	1.5 ± 0.1	1.6 ± 0.1
*τ*_v_ [ps]	32 ± 1	29 ± 1	40 ± 1	38 ± 1
*τ*_M_ [ns]	471 ± 19	486 ± 27	471^b^	510 ± 18
Δ*H*_M_ [kJ mol^−1^]	40 ± 4	37 ± 4	-	36 ± 1
*A*/*h* [10^6^ rad s^−1^]	−3.3 ± 0.1	−3.3 ± 0.1	-	−3.3 ± 0.1

^a^ The following parameters were fixed to appropriate values during the fitting procedure: *q* = 1; *r*_Gd-H_ = 3.0 Å; *a* = 4.0 Å; *D*^298^ = 2.24 × 10^−5^ cm^2^ s^−1^; *E*_V_ = 1 kJ mol^−1^; *E*_R_ = 10 kJ mol^−1^. ^b^ *τ*_M_ value determined for the mononuclear complex.

## Data Availability

The data are available on request from the corresponding author.

## Supplementary Materials

The following are available online, Equations used for fitting of ^1^H NMR profiles and ^17^O NMR relaxivity data Figures S1–S3: NMR spectra, Figures S4 and S5: ESI-MS spectra, Figure S6: transmetallation data. References [21,22,23,24,25,26,37,38,39] are cited in the Supplementary Materials.

## Sample Availability

Samples of the compounds are not available from the authors.
