# Supplementary material for: Towards Enhanced MRI Performance of Tumor-Specific Dimeric Phenylboronic Contrast Agents
Source: Molecules. 2021 Mar 19;26(6):1730. doi: 10.3390/molecules26061730 (PMC8003780; doi:10.3390/molecules26061730)
Supplement: Supplementary file 1 [file molecules-26-01730-s001.pdf]

Supplementary Material

Towards Enhanced MRI Performance of Tumor-Specific Dimeric Phenylboronic Contrast Agents

Jonathan Martinelli[1,2], Lorenzo Tei[2], Simonetta Geninatti Crich[3], Diego Alberti[3], and Kristina Djanashvili[1,*]

[1] Department of Biotechnology, Delft University of Technology, Van der Maasweg 9, 2629 HZ Delft, The Netherlands
[2] Department of Science and Technological Innovation, Università del Piemonte Orientale, Viale Michel 11, 15121 Alessandria, Italy
[3] Department of Molecular Biotechnology and Health Sciences, University of Torino, via Nizza 52, 10126 Torino, Italy

Fitting of ^{1}H NMRD profiles and ^{17}O NMR relaxivity data

From the two contributions (inner sphere ($r_{1,IS}$) and outer-sphere ($r_{1,OS}$)) to the longitudinal proton relaxation rate (r_1) (eq. 1), the former one is considered for the fitting of ^{1}H NMRD profiles [26]. The effect of outer-sphere water molecules in Gd^{III}-DOTA-EN-PBA analogues can be neglected.

$$r_1 = r_{1,IS} + r_{1,OS} \quad (1)$$

According to the Solomon–Bloembergen theory, the $r_{1,IS}$ is dominated by the dipolar interaction (eqs. 2 and 3), where q is the number of water molecules directly coordinated to the Gd-ion, T_{1M} is their longitudinal relaxation time, τ_M is the water-exchange correlation time [22-25].

$$r_{1,IS} = \frac{q}{55556(T_{1M}+\tau_M)} \quad (2)$$

$$\frac{1}{T_{1M}} = \frac{2}{15}\left(\frac{\mu_0}{4\pi}\right)^2 \frac{\hbar^2 \gamma_S^2 \gamma_I^2}{r_{GdH}^6} S(S+1)\left(\frac{3_{\tau d1}}{1+\omega_I^2\tau_{d2}^2}\right) \quad (3)$$

Here, r_{GdH} is the distance between the proton of a bound H_2O molecule and Gd-ion (fixed at 3.0 Å), $\mu_0/4\pi$ is the magnetic permeability in vacuum, S is the electron spin (7/2), γ_I and γ_S are the gyromagnetic ratios, and ω_I and ω_S are the Larmor frequencies of the proton and electron, respectively. T_{ie} (i = 1,2) represents electronic relaxation time that contributes to $\tau_{di}^{-1} = \tau_M^{-1} + \tau_R^{-1} + T_{ie}^{-1}$, expressed by eqs. 4 and 5 due to zero-field splitting (ZFS) [38].

$$\frac{1}{T_{1e}} = \frac{1}{25}\Delta^2\tau_V[4S(S+1)-3]\left(\frac{1}{1+\omega_S^2\tau_V^2} + \frac{1}{1+4\omega_S^2\tau_V^2}\right) \quad (4)$$

$$\frac{1}{T_{2e}} = \Delta^2\tau_V\left(\frac{5.26}{1+0.372\omega_S^2\tau_V^2} + \frac{7.18}{1+1.24\omega_S^2\tau_V^2}\right) \quad (5)$$

Here, Δ^2 represents the mean-squared fluctuation of the ZFS, τ_v is the correlation time for the instantaneous distortion of the coordination polyhedron of Gd-ion. The outer-sphere contribution to the relaxivity ($r_{1,OS}$) is described by eqs. 6 and 7, where N_A is the Avogadro's number, a_{GdH} the distance of diffusing water molecule in the closest approach to Gd-ion (fixed at 4.0 Å), D_{GdH} is diffusion coefficient and $J_{os}(\omega_S, Tje)$ (j = 1, 2) are spin density functions [25].

$$r_{1,IS} = \left(\frac{32\pi}{405}\right)\left(\frac{\mu_0}{4\pi}\right)^2 y_I^2 y_S^2 \hbar^2 S(S+1)\frac{N_A}{a_{GdH}D_{GdH}}[3J_{OS}(\omega_I, T_{1e}) + 7J_{OS}(\omega_S, T_{1e})] \quad (6)$$

$$J_{OS}(\omega_I, T_{je}) = Re\left\{\frac{1+\frac{1}{4}\left[i\omega\tau_{GdH}+\left(\frac{\tau_{GdH}}{T_{je}}\right)\right]^{\frac{1}{2}}}{1+\left[i\omega\tau_{GdH}+\left(\frac{\tau_{GdH}}{T_{je}}\right)^{\frac{1}{4}}\right]+\frac{4}{9}\left[i\omega\tau_{GdH}+\left(\frac{\tau_{GdH}}{T_{je}}\right)\right]+\frac{1}{9}\left[i\omega\tau_{GdH}+\left(\frac{\tau_{GdH}}{T_{je}}\right)^{\frac{1}{2}}\right]}\right\} \quad (7)$$

All correlation times τ (X = R, M, V) exhibit an exponential dependence (eq. 8), where E_X are activation energies (fixed at E_V = 1 kJ mol^{-1}, E_R = 1 kJ mol^{-1}) and R is the gas constant, leading to a temperature dependent diffusion coefficient D (fixed at 2.24×10^{-5} cm^2 s^{-1} for 298.15K).

$$\tau_X^T = \tau_X^{298} exp\left[\frac{E_X}{R}\left(\frac{1}{T} - \frac{1}{298.15}\right)\right] \quad (8)$$

$$D_{GdH}^T = D_{GdH}^{298} exp\left[\frac{E_D}{R}\left(\frac{1}{298.15} - \frac{1}{T}\right)\right] \quad (9)$$

Gd-induced water ^{17}O longitudinal and transverse relaxation rates ($1/T1r$ and$1/T_{2r}$) and angular frequencies ($\Delta_{\omega r}$) were calculated using eqs. 10 and 11 [38-40].

$$\frac{1}{T_{1r}} = \frac{1}{T_{1M}+\tau_M} \quad (10)$$

$$\frac{1}{T_{2r}} = \frac{1}{\tau_M}\frac{1}{T_{1M}+\tau_M} \quad (11)$$

NMR Spectra

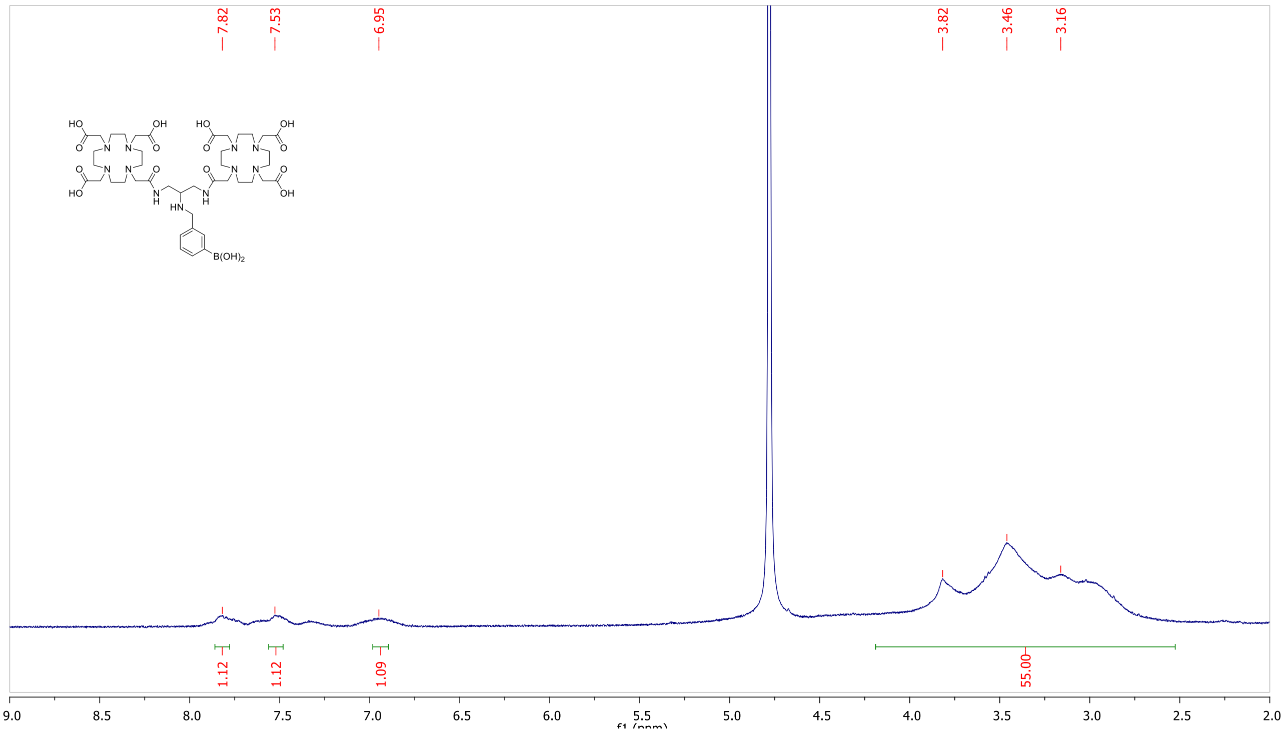

Figure S1. ^{1}H NMR spectrum in D_2O of (DOTA-EN)$_2$-PBA ligand.

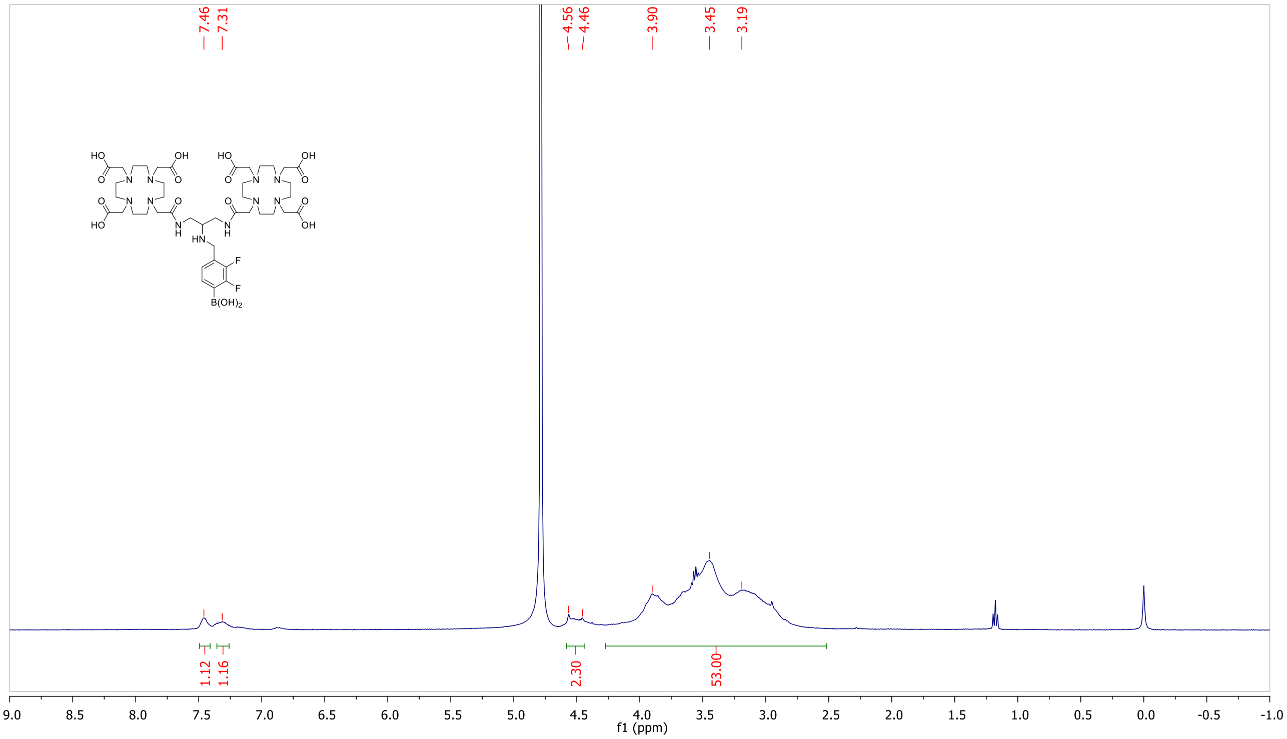

Figure S2. ^{1}H NMR spectrum in D_2O of (DOTA-EN)$_2$-F_2PBA ligand prepared *via* solid-phase synthesis. (residual Et_2O from precipitation is also observable).

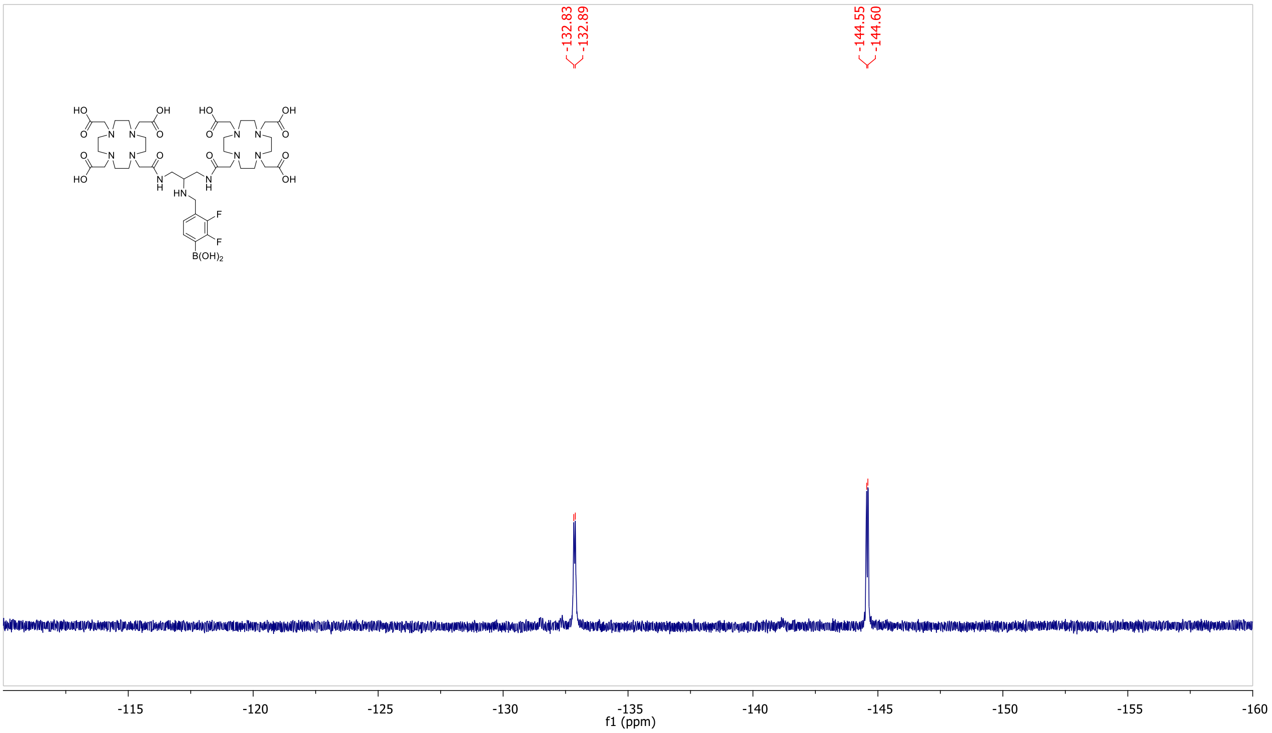

Figure S3. ^{19}F NMR spectrum in D_2O of (DOTA-EN)$_2$-F_2PBA ligand.

ESI-MS

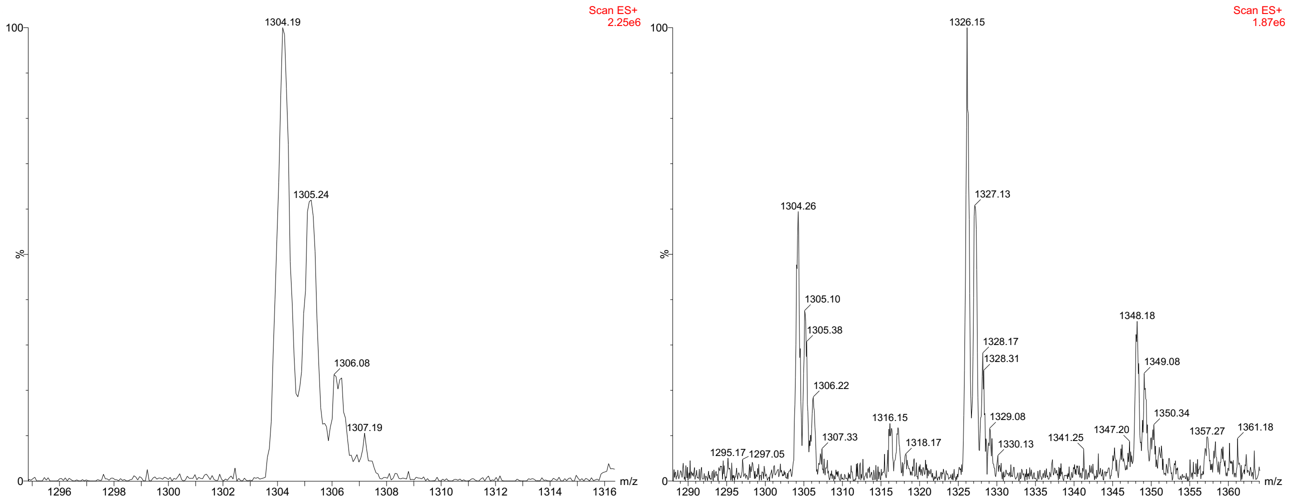

Figure S4. ESI$^+$ mass spectra of (Gd^{III}-DOTA-EN)$_2$-PBA (*left*) and with its mono- and di-sodium adducts (*right*).

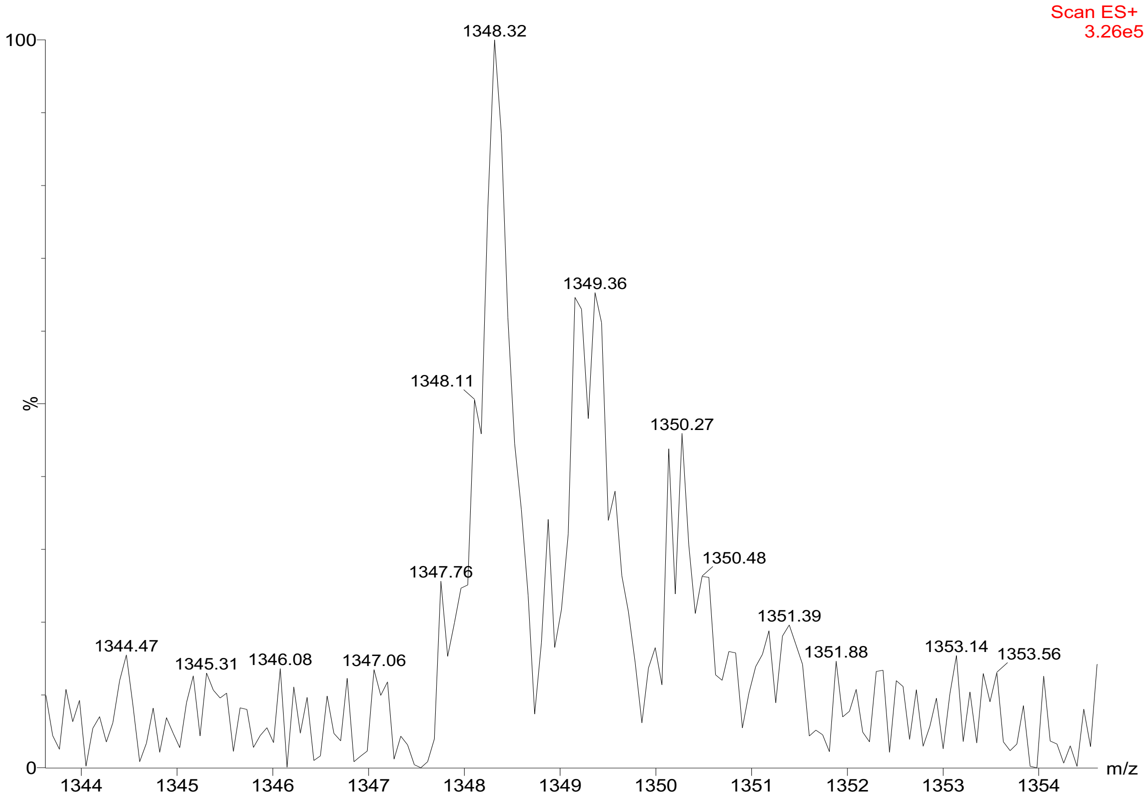

Figure S5. ESI$^+$ mass spectrum of (Gd^{III}-DOTA-EN)$_2$-F$_2$PBA.

Transmetallation study

Paramagnetic relaxation rate (R_1=1/T_1) of Gd^{III}-DOTA-EN-PBA (2.5 mM) was measured at 37° C in a phosphate buffer (pH=7) composed of KH_2PO_4 (0.025 M) and Na_2HPO_4 (0.04 M) in the presence of 2.5 mM of $ZnCl_2$ [9]. The reported R_1 values are corrected for diamagnetic contribution of the water relaxation. The measurements were performed at 400 MHz at 37° C in a Bruker Avance-400 NMR spectrometer in 5 mm sample tubes.

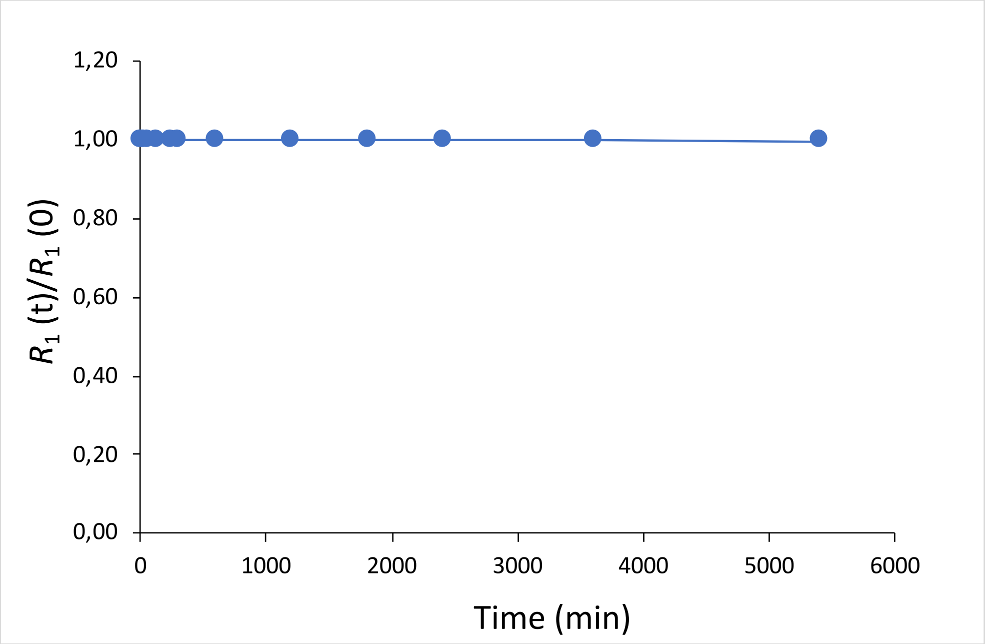

Figure S6. Evolution of the longitudinal relaxivity of Gd^{III}-DOTA-EN-PBA as a function of time time in the presence of competing Zn^{II}-ions in phosphate buffer. High ratio R_1(t)/R_1(0) (close to 1) indicates transmetallation high stability.
